# How the brain processes negative emotions

**DOI:** 10.7554/eLife.77550

**Published:** 2022-03-17

**Authors:** Gaowei Chen, Zijun Chen, Yingjie Zhu

**Affiliations:** 1 Institute of Brain Cognition and Brain Disease, Shenzhen Institutes of Advanced Technology, Chinese Academy of Sciences Shenzhen China

**Keywords:** parabrachial nucleus, thalamic paraventricular nucleus, affective states, anxiety, aversion, Mouse

## Abstract

A new brain circuit that contributes to aversive states, such as fear or anxiety, has been characterized in mice.

**Related research article** Zhu YB, Wang Y, Hua XX, Xu L, Liu MZ, Zhang R, Liu PF, Li JB, Zhang L, Mu D. 2022. PBN-PVT projection modulates negative affective states in mice. *eLife* **11**:e68372. doi: 10.7554/eLife.68372.

When an animal perceives a threat, this triggers an ‘aversive state’ in its brain, making the animal want to run away or hide from the threat. Aversive states are essential for survival, but not much is known about how the brain processes and modulates these negative emotions.

A region of the brainstem called the parabrachial nucleus (PBN) plays a critical role in encoding danger signals, such as threats (Campos et al., 2018). A recent study found that subpopulations of neurons in the PBN have different roles, and that their axons – which the neurons use to relay signals – project away from the PBN in distinct patterns (Chiang et al., 2020).

One of the projection targets of the PBN is the thalamic paraventricular nucleus (PVT), a brain region crucial for regulating emotional processing. The PVT is activated by various stressors (such as electric foot shock) and contributes to memories of fear (Do-Monte et al., 2015; Penzo et al., 2015; Zhu et al., 2016). However, even through our understanding of the neurocircuitry in the PVT has improved, the region of the brain that relays aversive information to the PVT is not well studied.

Now, in eLife, Ling Zhang and Di Mu, along with colleagues from Shanghai Jiao Tong University, Tongji University School of Medicine, Guangzhou Medical University and Southern University of Science and Technology – with Ya-Bing Zhu, Yan Wang and Xiao-Xiao Hua as joint first authors – report that, in mice, projections from the PBN into the PVT regulate aversive states, such as fear and anxiety (Zhu et al., 2022). Their observations describe important details of the previously uncharacterized brain circuit that connects the PBN and the PVT.

First, Zhu et al. used retrograde adeno-associated viruses to characterize the anatomic connections between the PBN and the PVT. This revealed that the neurons from the PBN that project into the PVT are located laterally (on the sides) in the PBN and can rarely be found medially (in the middle).

Next, Zhu et al. examined several genetic markers for subpopulations of PBN neurons from mice, and found that most of the PBN neurons that project into the PVT do not express these genes. This suggests that these cells are a newly identified population of PBN neurons.

To examine the functional connectivity between the PBN and the PVT, Zhu et al. expressed an optogenetic tool that allowed them to activate neurons in the PBN using light and record the synaptic response in PVT neurons. The results showed that PBN neurons send direct excitatory synaptic projections to the PVT. Zhu et al. also demonstrated that these synapses use glutamate as a neurotransmitter, since the synaptic responses can be completely blocked by a glutamate receptor antagonist.

Moreover, more connections were observed between the PBN and the medial and posterior (back) regions of the PVT than with the anterior (front) area of the PVT. Further viral tracing experiments also showed that the density of PBN axonal fibers was higher in the middle and posterior regions of the PVT, consistent with the notion that the posterior of the PVT is particularly sensitive to aversion (Gao et al., 2020).

To interrogate how important the pathway connecting the PBN to the PVT is to emotional processing, Zhu et al. used optogenetics (controlling neural activity with light) along with chemogenetics (controlling neural activity with chemicals). They found that activating the neurons of the PBN that project into the PVT with either light or chemicals induced instant aversion in mice: the animals exhibited fear-like and anxiety-like behaviors in the absence of a danger signal.

These results indicate that the pathway between the PBN and the PVT contributes to negative emotion processing. The next question to be answered is whether blocking this pathway would reduce negative emotions. Zhu et al. tested this hypothesis with optogenetics, using light to inhibit the projections between the PBN and the PVT in mice. When these mice were subjected to an electrical shock or the odor of a predator, they had reduced aversion-like and fear-like freezing behaviors. This establishes the pathway between the PBN and the PVT as a neural circuit essential to modulating negative emotion processing.

Consistent with previous findings, Zhu et al. showed that neurons in the PVT increased their activity after mice were subjected to two methods for inducing stress: mild electrical shocks and blowing puffs of air onto the face (Do-Monte et al., 2015; Penzo et al., 2015; Zhu et al., 2018). Based on the excitatory projections from the PBN to the PVT, this strongly implies that the aversive signal might in part originate in the PBN.

To confirm this, Zhu et al. stained cells in the PVT for Fos protein and mRNA, which will show up when a neuron has been active, and used optoelectrodes to record neural activity. The results showed that electric shocks activated broadly the same neurons in the PVT that became activated when the PBN was stimulated using optogenetics. Further optoelectrode recording results showed that stimulation of PBN inputs could increase activity in the PVT in response to aversive stimuli. In a final step, Zhu et al. used an anterograde labeling strategy, in which they traced axons from their source to their termination, to locate PVT neurons that received PBN inputs. When they directly activated these postsynaptic PVT neurons, the mice exhibited anxiety-like behaviors but not obvious pain-related behaviors.

The results of Zhu et al. address the question of where aversive signals processed by the PVT come from, indicating that inputs from the PBN may be critical for this process (Figure 1). The neurons from the PBN that feed into the PVT do not express current known PBN markers, which means they are a new subpopulation of neurons that will need to be further characterized, particularly at the molecular level. This will allow scientists to better understand how the brain processes and responds to threats, and how behaviors in response to threat evolved.

**Figure 1.**
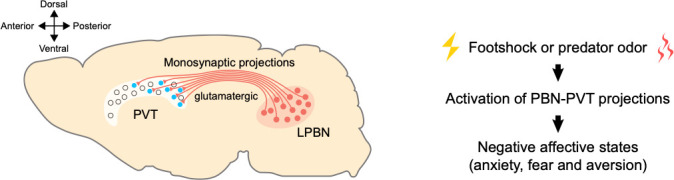
A pathway between the brainstem and the thalamus contributes to negative emotional processing. Left: neurons that produce glutamate (shown in red) in the lateral parabrachial nucleus (LPBN) of the brainstem send projections to the paraventricular thalamic nucleus (PVT). The PVT neurons that receive PBN inputs (shown in blue) are mainly located at medial and posterior PVT. The cross on the top left indicates where the anterior, posterior, ventral and dorsal parts of the brain lie, with medial parts in the middle. Right: external aversive stimuli (such as footshock or predator odor) activate the pathway from the PBN to the PVT, inducing negative affective states, such as anxiety, fear, and aversion.
